# Chronic postsurgical pain in children and young adults with cancer and choice of regional anesthesia for amputation and limb‐sparing surgery

**DOI:** 10.1002/cnr2.1719

**Published:** 2022-10-07

**Authors:** Doralina L. Anghelescu, Emma Johns, Shalini Bhatia, Michael J. Frett, Zhaohua Lu

**Affiliations:** ^1^ St. Jude Children's Research Hospital Memphis Tennessee USA

**Keywords:** amputation, chronic pain, neuropathic, oncology, pediatric, post‐surgical

## Abstract

**Background:**

Patients undergoing limb amputation (LA) or limb‐sparing (LS) for lower extremity oncologic diagnoses are at similar risk for chronic postsurgical pain of neuropathic nature (CPSP/NP). Regional anesthesia (RA) techniques are pre‐emptive measures to prevent the occurrence of the CPS/NP. However, recommendations for epidural (EP) versus peripheral nerve blocks (PNBs) lack in pediatric literature.

**Aims:**

This study investigates the incidence and duration of CPSP/NP and describes NP‐directed regimens.

**Methods and Results:**

Data on demographics, use of EP or PNB, duration of CPSP/NP, and NP‐directed medication were retrospectively collected for LA and LS between 2009 and 2019. Mixed effects logistic regression was used to compare the odds of CPSP/NP between EP and PNB. Cox PH model with adjustment for clustering due to multiple surgeries on patients was used to quantify rate of pain relief between surgery groups (LA vs. LS) and RA groups (EP vs. PNB). The incidence of CPSP/NP was 36 (23.8%) after 165 surgeries (150 patients). The odds of CPSP/NP after PNB were 2.5 times those of CPSP/NP after EP (*p* = .11). The rate of pain relief at any instant after the EP was 1.2 times that after PNB (*p* = .3). The rate of pain relief for LS with EP was 1.9 times that of pain relief for LA with EP, a statistically significant difference (*p* = .03). Gabapentin was used (94.5%), with addition of amitriptyline (24.2%) and both amitriptyline and methadone (12.7%).

**Conclusion:**

The LS with the EP group had a significantly higher rate of relief of CPSP/NP than LA with EP. Odds of CPSP/NP after PNB were 2.5 times those of CPSP after EP.

## 1 INTRODUCTION

Malignancies of the lower extremities are treated with complex modalities such as chemotherapy and definitive surgery by limb‐sparing (LS) or limb amputation (LA). Although LS procedures are generally favored due to higher functional outcomes, both procedures elicit neuropathic pain (NP) of comparable incidence and duration.^1^, ^2^ NP is described as tingling, burning, “pins and needles” sensation, shooting pain, and phantom limb pain.^1^, ^3^, ^4^, ^5^ Even though NP has been described in other pediatric populations, children with cancer are at greater risk of developing NP after surgery because of the additional contributing factor of exposure to chemotherapy.^3^, ^6^ Chronic postsurgical pain (CPSP), of duration ≥3 months, is estimated to affect children with 20% prevalence at 12 months,^7^ and thus represents a problem in pediatric populations.^1^, ^4^, ^8^ When pain of neuropathic nature (CPSP/NP) occurs, it can reduce the quality of life.^5^

Predictive factors in the development of CPSP include presurgical pain intensity, child/parent psychosocial factors, and severity of acute postoperative pain.^7^, ^8^, ^9^, ^10^ Efforts to prevent CPSP/NP include anesthetic plans aiming to establish pre‐emptive analgesia. Regional anesthesia techniques are pre‐emptive measures aimed to prevent immediate postoperative pain and potentially reduce the risk of the CPSNP. The safety and efficacy of neuraxial and perineural analgesia have been recognized in children.^11^, ^12^ However, recommendations for epidural (EP) versus peripheral nerve blocks (PNBs) lack in pediatric literature.^8^, ^11^ Multimodal perioperative analgesia involves “layering” of regional anesthesia (RA), opioids, non‐opioids, and NP‐directed medications such as gabapentinoids; nevertheless, consensus on optimal regimens is lacking.^3^, ^8^, ^13^

In this study, we retrospectively studied the associations between RA (EP or PNB) and the incidence and duration of CPSP/NP after LS and LA/disarticulations for lower extremity malignant tumors. The specific aims were to characterize pain outcomes including the prevalence and probability of CPSP/NP, the duration of CPSP/NP (days), and the NP‐directed medications (gabapentinoids, tricyclic antidepressants, and methadone), as doses (mg/kg/day), and duration (days).

## 2 METHODS

With approval from the Institutional Review Board, all the patients who underwent LS or LA disarticulation surgery for lower extremity malignancies between January 1, 2009, and July 1, 2019 were included in this single‐institution retrospective review from St. Jude Children's Research Hospital (Memphis, TN, United States). Data pertaining to all their surgeries, either LS, or LA, were eligible for analysis; therefore, each patient may have contributed with data pertaining to several sequential surgeries in the LS and LA categories. There were no exclusion criteria in this retrospective study design. Data collected included demographic characteristics (age, gender, race, ethnicity, weight), oncological diagnosis, surgical procedure, type of RA, and long‐term pain outcomes. Types of RAs included EP continuous infusion, continuous PNB, and single‐dose PNB. Intra‐anesthetic records, surgical records, and nursing intraoperative records were compared to ensure data accuracy.

Data on pain outcomes were collected from pain service notes and included the following: (1) duration (days) of postsurgical pain that met characteristics of NP, (2) duration (days) of therapy for each line of NP‐targeted medication, and (3) initial and maximum dose (mg/kg/day). Descriptors used to characterize NP included tingling, burning, shooting, “pins and needles,” and phantom limb pain (PLP). The NP‐directed medications included gabapentinoids (gabapentin, pregabalin), tricyclic antidepressants (amitriptyline), and methadone. The NP treatment follows an institutional algorithm that escalates medications in successive “layers” of gabapentinoids, tricyclic antidepressants, and methadone, based on tolerance and analgesic efficacy. Pain was considered resolved if all NP medications were weaned without recurrence of NP symptoms. Patients were considered lost to follow up due to transfer of care to another institution, death before complete resolution of the pain, or weaning off NP‐targeting medications, or due to missing documentation to support the resolution of NP. The pain outcomes were compared between groups treated with EP and PNB.

If a patient underwent a subsequent surgery while ongoing treatment for postsurgical NP (e.g., LA/disarticulation after an initial LS while having ongoing postsurgical NP), pain outcomes were attributed to the subsequent surgery. Patients with CPSP/NP were defined as being treated postoperatively with NP‐targeted lines of medications for longer than 90 days.

### 2.1 Statistical analysis

Descriptive statistics were reported as frequency (percent) and mean (SD). Duration of CPSP/NP was calculated as the time from surgery to complete resolution of NP and absence of NP‐directed medications.

The rate of pain relief at any instance was compared between EP or PNB groups and between surgery (LA or LS) and treatment (EP or PNB) combination groups, using the Cox PH model with adjustment for clustering due to multiple surgeries on patients. CPSP/NP was defined as postsurgical pain of duration more than 90 days, and mixed effects logistic regression was used to compare the odds of CPSP/NP between RA groups. All the analyses were performed in R (version: 3.6.2). All *p* < .05 were considered significant.

## 3 RESULTS

### 3.1 Patient characteristics

Of 172 surgeries performed for LS, LA, or disarticulation during the study period, 7 were excluded due to missing data and 165 surgeries (150 unique patients) contributed to data analyses. The study included 86 (57.3%) males; mean (SD) age 12.5 (3.9) years; 112 (74.7%) White. One hundred thirty‐seven (91.3%) patients underwent one, 11 (7.3%) underwent two, and two (1.3%) underwent three surgeries (i.e., LS followed by LA and disarticulation). A RA technique was utilized intraoperatively and postoperatively in 162 (98.2%) of LS and LA disarticulations surgeries. Ninety‐four (57%) surgeries were completed with PNB (91 continuous infusions and 3 single dosing), 68 (41.2%) were completed with EP, and 3 (1.8%) had none. Forty‐four (26.7%) patients had LA and 120 (72.7%) had LS surgeries. Twenty‐six (15.8%) patients had LA with EP, 42 (25.5%) LS with EP, 16 (9.7%) LA with PNB, 77 (46.7%) LS with PNB, and 4 (2.4%) patients did not have information regarding RA and surgery type combination (Tables 1 and 2).

**TABLE 1:** Patient demographic and clinical and surgical characteristics

Demographics	Study group
Number of patients	(*N* = 150)
*Sex*	
Male	86.0 (57.3%)
Female	64.0 (42.7%)
*Age* (years)	
Mean (SD)	12.5 (3.9)
Median [Min, Max]	13.0 [3.0, 21.0]
*Race*	
White	112.0 (74.7%)
Black	28.0 (18.7%)
Black and White	4.0 (2.7%)
Multiple Race (NOS)	3.0 (2.0%)
Asian	1.0 (0.7%)
Asian and White	1.0 (0.7%)
Other	1.0 (0.7%)
*Ethnicity*	
Non‐Spanish Speaking, Non‐Hispanic	121.0 (80.7%)
NOS Spanish/Hispanic/Latino	11.0 (7.3%)
South or Central American	7.0 (4.7%)
Mexican/Chicano	6.0 (4.0%)
Puerto Rican	4.0 (2.7%)
Caribbean	1.0 (0.7%)

Surgeries characteristics	(*N* = 165)
*Regional anesthesia*	
PNB^a^	94.0 (57%)
EP	68.0 (41.2%)
None	3.0 (1.8%)
*Post‐surgical pain status*	
Followed—Pain resolved	141.0 (85.5%)
Lost to Follow Up^b^	24.0 (14.5%)
*Number of surgeries*	
1	137.0 (91.3%)
2	11.0 (7.3%)
3	2.0 (1.3%)
*Surgery type*	
LS	44.0 (26.7%)
LA	120.0 (72.7%)
Missing	1.0 (0.6%)
*Surgery type and regional anesthesia*	
LA and EP	26.0 (15.8%)
LS and EP	42.0 (25.5%)
LA and PNB	16.0 (9.7%)
LS and PNB	77.0 (46.7%)
Missing	4.0 (2.4%)

Abbreviations: EP, epidural; LA, limb amputation; LS, limb sparing; Max, maximum; Min, minimum; NOS, not otherwise specified; PNB, peripheral nerve block; SD, standard deviation.

^a^ 91 continuous infusions and 3 single dosing.

^b^ Of 24 lost to follow up before resolution of neuropathic, 14, 7, and 3 were lost between 1 and 90 days, 91 and 182 days, and after 183 days, respectively.

**TABLE 2:** Distribution of patients with chronic postsurgical pain of neuropathic nature for more than 90 days^a^

CPSP/NP	Peripheral nerve block (PNB) (*N* = 84)	Epidural (*N* = 64)	None (*N* = 3)	Study group^a^ (*N* = 151)
No	59.0 (70.2%)	53.0 (82.8%)	3.0 (100.0%)	115.0 (76.2%)
Yes	25.0 (29.8%)	11.0 (17.2%)	0.0 (0.0 %)	36.0 (23.8%)

Abbreviation: CPSP, chronic postsurgical pain of neuropathic nature.

^a^ 151 surgeries were included in analysis, after exclusion of 14 surgeries after which patients were lost to follow up for pain assessment within the first 90 days (therefore not meeting the chronic pain criterion).

### 3.2 Incidence, probability, and duration of CPSP/NP

The calculation of incidence of CPSP/NP was based on 151 surgeries with evaluable data. Data from mixed‐effects logistic regression analysis were excluded for 14 surgeries, after which patients were lost to follow up before 90 days (therefore not meeting the chronic pain criterion). Thirty‐six (23.8%) surgeries resulted in CPSP/NP. No difference was found in odds of CPSP/NP between EP and PNB (*p* = .11). Odds of having CPSP/NP after PNB were 2.5 (0.8–7.9) times the odds after EP. No difference was found in pain relief between EP and PNB groups (*p* = .3). Rate of pain relief at any instance in the EP group was 1.2 times that of the PNB group. There was a difference in the rate of pain relief between LS with EP and LA with EP (*p* = .03); rate of pain relief in LS with EP was 1.9 times that in LA with EP. Among all 165 surgeries, mean (SD) number of days patients had postsurgical pain/NP were 60.8 (61.1) overall, 66.6 (60.7) in PNB group, and 54.7 (62.1) in EP group (Table 3).

**TABLE 3:** Duration of postsurgical pain of neuropathic nature

Time (days)	Peripheral nerve block (*N* = 94)	Epidural (*N* = 68)	None (*N* = 3)	Study group (*N* = 165)
Mean (SD)	66.6 (60.7)	54.7 (62.1)	17.3 (2.1)	60.8 (61.1)
Median [Min, Max]	46.5 [2.0, 319.0]	35.0 [3.0, 302.0]	18.0 [15.0, 19.0]	39.0 [2.0, 319.0]

### 3.3 NP‐specific medications—Type, duration, and dose

Data for all 165 surgeries were used in analysis of medications. Patients received gabapentin in 156 (94.5%) surgeries (Table 4). Among them, 65 (41.7%) received EP and 89 (57.1%) received PNB. Seven (4.2%) patients received pregabalin as a rotation from gabapentin: 2 had PNB and 5 had EP. After 40 (24.2%) surgeries, patients also required amitriptyline: 19 (47.6%) had PNB and 21 (52.4%) had EP. Methadone was administered in 21 (12.7%) patients: 11 (52.6%) had PNB and 10 (47.6%) had EP.

**TABLE 4:** Postoperative treatment with neuropathic pain medications and type of intra‐operative regional anesthetic

	Peripheral nerve block (*N* = 94)	Epidural (*N* = 68)	None (*N* = 3)	Study group (*N* = 165)
*Gabapentin*				
No	5.0 (5.3%)	3.0 (4.4%)	1.0 (33.3%)	9.0 (5.5%)
Yes	89.0 (94.7%)	65.0 (95.6%)	2.0 (66.7%)	156.0 (94.5%)
*Pregabalin*				
No	92.0 (97.9%)	63.0 (92.6%)	3.0 (100.0%)	158.0 (95.8%)
Yes	2.0 (2.1%)	5.0 (7.4%)	0.0 (0.0%)	7.0 (4.2%)
*Amitriptyline*				
No	75.0 (79.8%)	47.0 (69.1%)	3.0 (100.0%)	125.0 (75.8%)
Yes	19.0 (20.2%)	21.0 (30.9%)	0.0 (0.0%)	40.0 (24.2%)
*Methadone*				
No	83.0 (88.3%)	58.0 (85.3%)	3.0 (100%)	144.0 (87.3%)
Yes	11.0 (11.7%)	10.0 (14.7%)	0.0 (0.0%)	21.0 (12.7%)

Mean (SD) number of days patients received gabapentin in the PNB group was 69.3 (60.9), and in the EP group it was 56.6 (62.5) (Table 5). Mean (SD) number of days patients received pregabalin was 141 (125) in PNB and 268 (416) in the EP group. Mean (SD) number of days patients received amitriptyline in the PNB group was 61.4 (64.3), and in the EP group it was 59.5 (77.2). Mean (SD) number of days patients received methadone was 70.6 (60.8) and 110 (85) in the PNB and EP groups, respectively.

**TABLE 5:** Duration of treatment with neuropathic pain medications

	Peripheral nerve block (*N* = 94)	Epidural (*N* = 68)	None (*N* = 3)	Study group (*N* = 165)
*Days on Gabapentin*				
Mean (SD)	69.3 (60.9)	56.6 (62.5)	16.1 (2.2)	63.3 (61.5)
Median [Min, Max]	48.5 [4.0, 319.0]	35.7 [2.6,302]	16.1 [14.6, 17.6]	45.6 [2.6, 319.0]
Not treated	5.0 (5.3%)	3.0 (4.4%)	1.0 (33.3%)	9.0 (5.5%)
*Days on Pregabalin*				
Mean (SD)	141.0 (125.0)	268.0 (416.0)	NA (NA)	232.0 (349.0)
Median [Min, Max]	141.0 [52.6, 230.0]	82.5 [34.6,1010.0]	NA [NA, NA]	82.5 [34.6,1010.0]
Not treated	92.0 (97.9%)	63.0 (92.6%)	3.0 (100%)	158.0 (95.8%)
*Days on Amitriptyline*				
Mean (SD)	61.4 (64.3)	59.5 (77.2)	NA (NA)	60.4 (70.5)
Median [Min, Max]	36.0 [1.0, 245.0]	39.0 [8.0,357.0]	NA [NA, NA]	37.5 [1.0, 357.0]
Not treated	75.0 (79.8%)	47.0 (69.1%)	3.0 (100%)	125.0 (75.8%)
*Days on Methadone*				
Mean (SD)	70.6 (60.8)	110.0 (85.0)	NA (NA)	89.5 (74.3)
Median [Min, Max]	71.0 [5.0, 232.0]	98.0 [8.0, 246.0]	NA [NA, NA]	71.0 [5.0, 246.0]
Not treated	83.0 (88.3%)	58.0 (85.3%)	3.0 (100.0 %)	144.0 (87.3%)

Mean (SD) initial dosage (mg/kg/day) for gabapentin was 16 (6.7) in the PNB group, and 18.3 (11.7) in the EP group (Table 6). Mean (SD) maximum dosage (mg/kg/day) of gabapentin in the PNB group was 39.7 (20.8) and in the EP group it was 37.9 (22.3). Pregabalin mean dose (SD) (mg/kg/day) was used in the PNB group as 3.5 (0.2) and in the EP group as 5.6 (2.4). Mean (SD) of initial dose (mg/kg/day) amitriptyline was 0.4 (0.2) and 0.5 (0.2) in PNB and EP groups, respectively. Mean (SD) of maximum dose (mg/kg/day) amitriptyline was 0.7 (0.2) and 1 (0.4) and in the PNB and EP groups, respectively. Mean (SD) of initial dose methadone (mg/kg/day) was 0.2 (0.1) and 0.3 (0.1) in PNB and EP groups, respectively. Mean (SD) of maximum dose methadone (mg/kg/day) was 0.3 (0.1) and 0.5 (0.2) in PNB and EP groups, respectively.

**TABLE 6:** Initial & maximum dosing regimens for neuropathic pain treatment

	Peripheral nerve block (*N* = 94)	Epidural (*N* = 68)	None (*N* = 3)	Study group (*N* = 165)
*Gabapentin: INITIAL dosing regimen* (mg/kg/day)				
Mean (SD)	16.0 (6.7)	18.3 (11.7)	25.9 (10.9)	17.1 (9.2)
Median [Min, Max]	14.7 [5.4, 40.3]	15.2 [6.2, 68.4]	25.9 [18.2, 33.6]	14.8 [5.4, 68.4]
Not treated	5.0 (5.3%)	3.0 (4.4%)	1.0 (33.3%)	9.0 (5.5%)
*Gabapentin: MAX dosing regimen* (mg/kg/day)				
Mean (SD)	39.7 (20.8)	37.9 (22.3)	25.9 (10.9)	38.7 (21.4)
Median [Min, Max]	35.4 [7.0, 98.9]	33.3 [7.9, 90.0]	25.9 [18.2, 33.6]	34.4 [7.0, 98.9]
Not treated	5.0 (5.3%)	3.0 (4.4%)	1.0 (33.3%)	9.0 (5.5%)
*Pregabalin dosing regimen* (mg/kg/day)				
Mean (SD)	3.5 (0.2)	5.6 (2.4)	NA (NA)	5.098 (2.2)
Median [Min, Max]	3.5 [3.4, 3.7]	6.3 [2.0, 8.2]	NA [NA, NA]	4.5 [2.0, 8.2]
Not treated	92.0 (97.9%)	63.0 (92.6%)	3.0 (100.0%)	158.0 (95.8%)
*Amitriptyline: INITIAL dosing regimen* (mg/kg/day)				
Mean (SD)	0.4 (0.2)	0.5 (0.2)	NA (NA)	0.5 (0.2)
Median [Min, Max]	0.4 [0.2, 0.9]	0.1 [0.2, 1.1]	NA [NA, NA]	0.4 [0.2, 1.1]
Not treated	75.0 (79.8%)	47.0 (69.1%)	3.0 (100.0%)	125.0 (75.8%)
*Amitriptyline: MAX dosing regimen* (mg/kg/day)				
Mean (SD)	0.7 (0.2)	1.0 (0.4)	NA (NA)	0.8 (0.4)
Median [Min, Max]	0.7 [0.3, 1.0]	0.9 [0.3, 1.8]	NA [NA, NA]	0.8 [0.3, 1.8]
Not treated	75.0 (79.8%)	47.0 (69.1%)	3.0 (100.0%)	125.0 (75.8%)
*Methadone: INITIAL dosing regimen* (mg/kg/day)				
Mean (SD)	0.2 (0.1)	0.3 (0.1)	NA (NA)	0.2 (0.1)
Median [Min, Max]	0.2 [0.1, 0.3]	0.2 [0.2, 0.5]	NA [NA, NA]	0.2 [0.1, 0.5]
Not treated	83.0 (88.3%)	3.0 (100.0%)	3.0 (100.0%)	144.0 (87.3%)
*Methadone: MAX dosing regimen* (mg/kg/day)				
Mean (SD)	0.3 (0.1)	0.5 (0.2)	NA (NA)	0.4 (0.2)
Median [Min, Max]	0.3 [0.1, 0.4]	0.5 [0.2,1.1]	NA [NA, NA]	0.3 [0.1, 1.1]
Not treated	83.0 (88.3%)	58.0 (85.3%)	3.0 (100.0%)	144.0 (87.3%)

## 4 DISCUSSION

Our study specifically examined long‐term pain outcomes in pediatric and young adult patients with cancer, with focus on CPSP with NP characteristics and its associations with types of RA. An NP diagnosis is based on partial or complete sensory loss in a defined neuroanatomical area, with paradoxical hypersensitivity (allodynia and hyperalgesia), in the context of history of disease or lesion of the nervous system.^5^ The incidence of 23.8% is, not surprisingly, among the highest reported, as LS and LA disarticulations are inherently highly traumatic to the peripheral nervous system and CPSP occurs at higher rates after invasive surgeries in densely innervated areas. In a systematic review and meta‐analysis, CPSP had an estimated prevalence of 20% in pediatric surgeries,^7^ while a prospective observational study reported a pediatric CPSP prevalence of 10.9%, but of note, mostly of neuropathic origin (64.3%).^10^ In adult studies, acute postsurgical pain resulted in CPSP in 10%–50% of cases and became severe in 2%–10% of cases.^5^

Furthermore, we described the odds of CPSP/NP following PNB as being 2.5 times that of pain following EP, and the rate of pain relief in the EP group as being 1.2 times that of the PNB group (while not statistically significant). A statistically significant difference was found between the rate of pain relief in the LS with EP and LA with EP groups, with twice as high the rate of pain relief in the LS with EP group. The fact that LS with EP had the shortest duration of pain (29 days) may suggest better prognosis of pain relief over time with EP as a favored modality for LS. We reported a lower incidence of CPSP/NP in the EP versus the PNB groups, which may reflect clinical relevance, despite the lack of statistical significance.

The increased focus on approaches to prevent and treat post‐surgical NP, even more important than the focus on nociceptive pain, is justified by concerns that although NP may be less prevalent than nociceptive pain in pediatric oncology, its treatment is more difficult and requires a longer follow up and complex pharmacological and nonpharmacological management.^14^ Clinicians should be aware of the possibility of CPSP/NP and assess for NP characteristics, because distinct NP‐directed lines of medication are necessary.^15^ A prospective study of 37 patients (38 surgeries) after osteosarcoma‐directed LS or LA found NP in 81.6%, with comparable duration after LS or LA.^1^ This high incidence may reflect particular vulnerabilities and predisposition to developing NP due to chemotherapy, pain pre‐existent prior to surgery, and surgical trauma.^6^ Phantom limb pain (PLP) is better characterized than post–limb‐sparing pain, and is described as CPSP with complex pathophysiology and proposed central and peripheral mechanisms,^3^, ^16^ with onset soon after surgery and persistence of months to years.^3^ In pediatric oncology, the prevalence of PLP varies between 48% and 90%.^3^, ^4^, ^6^ Of pediatric cancer‐related amputees, 76% experienced PLP in the first year, consistent with the incidence established by earlier studies.^17^

Although the role of EP in postoperative pain relief is well established, effectiveness of PLP prevention is inconsistently reported.^18^, ^19^ Preoperative epidural blockade 18 h before amputation did not prevent PLP during the first year postoperatively.^20^ Conversely, others showed reduced incidence of PLP after amputation with epidural analgesia.^21^, ^22^, ^23^ Epidural analgesia for 48 h preoperatively was associated with decreased PLP at 6 months.^24^ Preemptive epidural analgesia 72 h preoperatively and 72 h postoperatively reduced the incidence of PLP during the first year compared to treatment with opioids and NSAIDs.^25^ Overall, studies found that preoperative EP analgesia had a beneficial impact on acute and chronic pain, although the statistical impact is inconclusive for EP as a PLP preventative therapy.^18^, ^23^

In adults undergoing lower extremity LA, preemptive use of continuous PNB lowered the postoperative incidence of pulmonary complications, acute postoperative pain scores, and opioid use when compared to general anesthesia.^26^ Continuous infusions are preferred to single dosing techniques.^27^ Perineural anesthesia may show beneficial results in preventing PLP after LA.^7^, ^27^, ^28^, ^29^, ^30^ A higher incidence of PLP after LA was found after general anesthesia than neuraxial anesthesia or PNB.^31^ However, other studies showed no benefit with PNB use for PLP prevention.^32^, ^33^, ^34^ A systematic review and meta‐analysis indicated that PNB did not reduce PLP, but the GRADE quality evidence was very low^34^; overall, the current evidence for efficacy in PLP prevention is limited and inconsistent.

Direct comparisons between EP and PNB techniques are limited. Both provide adequate postoperative pain relief and opioid‐sparing effects. Reviews by Von Plato et al. and Ahuja et al. summarized current evidence on RA techniques after major LA.^18^, ^19^ A randomized prospective trial provided direct comparison and reported no difference in long‐term outcomes between EP and continuous PNB.^35^ Specifically, EP started 24 h preoperatively was not superior to PNB in preventing PLP.^35^ Studies related to RA are heterogeneous in methodology and results, preventing the selection of a consistent therapy for prevention and treatment of PLP.^18^, ^19^, ^23^, ^36^ Although prospective controlled trials are recommended to define recommendations for clinical practice, the choice of RA is currently mostly based on clinicians' preference. Our analysis in the pediatric and young adult oncologic population showed results similar to those of Lambert et al., lacking statistical significance in the difference between EP and PNB for the incidence of CPSP/NP.^35^ However, the fact that the LS group treated with EP showed a statistically significant lower duration of pain can provide direction for clinical decisions when RA interventions are considered.

Literature lacks conclusions about whether RA could decrease the incidence of CPSP in LA.^37^ A review of orthopedic oncologic surgeries indicated that whereas RA may be effective postoperatively, pain management requires multimodal approaches.^12^ The RA techniques within multimodal plans contribute to reducing severe immediate postoperative pain, a consistent risk factor for CPSP.^18^, ^19^, ^35^, ^36^, ^38^ Optimized analgesia in the acute phase after LA is theorized to help prevent chronic pain. In surgeries for patients with high risk of chronic pain development (LA, breast cancer surgery, thoracotomies, Cesarean sections), RA initiation is recommended prior to incision and continued 24–72 h as preventative strategy for postoperative pain and CPSP.^37^ Cochrane reviews support RA as reducing CPSP after breast cancer surgery, thoracotomies, and Cesarean sections; however, this conclusion was not extended to LA due to variable timing of RA administration and heterogenous study designs.^39^, ^40^, ^41^ A review of 39 randomized controlled trials found the strongest evidence for use of EP for thoracotomies and paravertebral blocks for breast surgery.^39^ Adjuvants like ketamine in combination with perioperative neural blockades warrant further investigation in CPSP prevention.^5^

Overall, the current literature comparing RA modalities is limited to specific types of surgery for adults. It is important to note the paucity of data in pediatric patients: although RA in children is safe and effective for postoperative pain, little is known about its role in long‐term pain outcomes.^11^

When characterizing the NP‐targeted medications dosing, we found that a higher percentage of patients received gabapentin, pregabalin, amitriptyline, and methadone in the EP than the PNB group, and greater dosages were used. Our approach to NP treatment is based on a clinical algorithm, including anticonvulsants (gabapentin and pregabalin), tricyclic antidepressants (TCA; amitriptyline), and methadone added in a stepwise manner. One in three patients can be expected to require an escalation of the regimen, with addition of amitriptyline and/or methadone to gabapentinoids.^1^ In our study, 24.2% of NP treatment regimens included amitriptyline in addition to gabapentinoids, and 12.7% required addition of methadone to gabapentinoids and amitriptyline.

To establish preemptive analgesia, in our practice, gabapentin is started 3–5 days prior to surgery, as 15 mg/kg/day divided into three doses. Preoperative gabapentin may be effective to reduce the incidence of CPSP.^13^, ^42^ A multimodal regimen for posterior spinal fusion used preoperative gabapentin as three daily doses of 5 mg/kg for age <12 and/or <40 kg, 200 mg for age >12 and 40–59 kg, and 300 mg for age >12 and >60 kg.^11^ Gabapentin, in combination with a postoperative opioid regimen, has been shown to effectively treat NP by improving analgesia, producing less side‐effects, and decreasing total pain scores.^43^, ^44^, ^45^ The pediatric literature suggests that dosing regimens of gabapentin for NP should start at 10 mg/kg/day and increase to 30 mg/kg/day over a few days to a week.^46^, ^47^, ^48^ Gabapentin may not achieve efficacy for NP treatment in the absence of dose escalation. A prospective randomized trial of gabapentin 20 mg/kg/day for vincristine‐related NP suggested lack of effect, possibly related to low dosing lacking escalation.^49^ The maximum dose generally accepted is 70 mg/kg/day and should not exceed 3600 mg/day,^13^ which is rarely achieved in children. In pediatric oncology, postsurgical NP was noted to be treated with higher gabapentin doses than those for chemotherapy‐related NP.^15^ Doses of 45 mg/kg/day are the highest reported in pediatrics, below the maximum adult dosages of 50–70 mg/kg/day.^1^, ^11^ Our study findings are consistent with the low dosing regimens noted before, with escalation to a mean (SD) maximum of 38.7 (21.4) mg/kg/day. When gabapentin is not efficacious, pregabalin is an alternative, titrated to 5 mg/kg/day, starting at 75 mg and escalating to 150–300 mg.^15^ Our study indicated a pregabalin mean (SD) dose of 4.98 (2.19) mg/kg/day, consistent with this recommendation. In general, both anticonvulsants tend to be underdosed for NP in pediatrics; this could be attributed to the concurrent utilization of several lines of NP medication for achieving effective pain management at lower doses.^15^

Amitriptyline doses in our study as mean (SD) (mg/kg/day) were an initial and maximum of 0.472 (0.211) and 0.820 (0.369), respectively, and are among the higher values reported in literature, which start with 0.1 mg/kg and gradually increase to a maximum of 1 mg/kg at bedtime.^13^ Higher doses may suggest amitriptyline's utilization to mitigate sleep disorders, anxiety, and depression, all potential NP components.^47^, ^50^ As almost half of the postsurgical treatment combinations based on amitriptyline were escalated to include methadone (10 out of 21), our higher amitriptyline dosing than generally reported could reflect the persistence and difficulty of treating NP. The reported dosage of 0.35–0.40 mg/kg/day is lower than the anti‐depressive dose.^15^ Dosing of 10 or 20 mg twice daily (BID) is less favored due to the side effect of drowsiness.^50^

Our reported mean (SD) initial and maximum methadone dosing (mg/kg/day) of 0.214 (0.0951) and 0.362 (0.209), respectively, are consistent with previously reported dosing.^1^ The EP group received higher doses (mg/kg/day) than the PNB group. This third‐line therapy for NP, although rarely necessary, is started at 5–10 mg taken 2–3 times a day. A retrospective study of methadone in pediatric hematology/oncology reported starting doses of 0.06 to 3.8 mg/kg/day (median 0.32 mg/kg/day) to achieve analgesic efficacy in 40% of patients with NP.^51^ Methadone dosing of 0.06–32.7 mg/kg/day has been reported for NP treatment in pediatric oncology, including two cases with opioid tolerance requiring high doses of 24.8 and 32.7 mg/kg/day.^47^ Methadone is unique among opioids as an N‐methyl‐d‐aspartate receptor antagonist, which mediates the effectiveness for NP. There is no maximum dose; instead it can be titrated up (slowly, because of its long half‐life) until pain relief is achieved or side effects appear.^13^

### 4.1 Limitations and strengths

This study has some limitations. Although literature reflects the impact of psychosocial determinants on CPSP development more than biological determinants,^7^, ^8^, ^9^ our investigation did not explore beyond biological factors. When considering CPSP in view of modifiable and non‐modifiable factors, our study targeted a modifiable aspect, that of optimized analgesia through RA use. Our study lacked a homogenous distribution between LA and LS surgeries, because LS is preferred to LA to preserve functionality; nevertheless, based on previous studies, both oncologic surgeries behave similarly in terms of postsurgical pain.^1^ Furthermore, we acknowledge that the selection of RA technique was based on the preference of the anesthesiology team, reflecting their beliefs of superiority of one versus the other approach, as well as level of comfort with the selected technique. The retrospective nature of our study is also a limitation. Additionally, we did not investigate pain scores in the immediate postoperative period. Given that poorly controlled pain in the acute setting postoperatively can contribute to the development of CPSP, this information may have strengthened our findings. Long‐term follow up for pain was not possible in a subset of patients who were lost to follow up; nevertheless, our long duration of follow up strengthens the study and no prior study has analyzed as far longitudinally as we have.

## 5 CONCLUSION

The incidence of CPSP/NP in our population was 23.8% after LS and LA/disarticulation surgeries. LS surgeries with EP treatment had a significantly lower duration of pain compared to the other combinations of surgery and RA. The median duration of CPSP/NP of 38 days for EP versus 50 days for PNBs may have clinical significance, suggesting that EP interventions provide better prognosis of pain relief over time. Our study provides a comprehensive analysis of NP‐directed medication dosing regimens in CPSP/NP treatment, indicating higher dosing regimens than previously reported and providing a platform for development of standardized regimens. Prospective multi‐institutional studies are necessary to further elucidate the association of RA on CPSP in pediatric oncology and establish refined analgesic treatment regimens.

## AUTHOR CONTRIBUTIONS

**Doralina L. Anghelescu:** Conceptualization (lead); data curation (supporting); formal analysis (supporting); funding acquisition (supporting); investigation (lead); methodology (lead); project administration (supporting); resources (supporting); software (supporting); supervision (lead); validation (supporting); visualization (equal); writing – original draft (equal); writing – review and editing (equal). **Emma Johns:** Conceptualization (supporting); data curation (lead); formal analysis (supporting); funding acquisition (supporting); investigation (supporting); methodology (supporting); project administration (supporting); resources (supporting); software (supporting); supervision (supporting); validation (supporting); visualization (supporting); writing – original draft (equal); writing – review and editing (equal). **Shalini Bhatia:** Conceptualization (supporting); data curation (supporting); formal analysis (lead); funding acquisition (supporting); investigation (supporting); methodology (supporting); project administration (supporting); resources (supporting); software (lead); supervision (supporting); validation (supporting); visualization (supporting); writing – original draft (supporting); writing – review and editing (supporting). **Michael J. Frett:** Conceptualization (supporting); data curation (supporting); formal analysis (supporting); funding acquisition (supporting); investigation (supporting); methodology (supporting); project administration (supporting); resources (supporting); software (supporting); supervision (supporting); validation (supporting); visualization (supporting); writing – original draft (supporting); writing – review and editing (supporting). **Zhaohua Lu:** Conceptualization (supporting); data curation (supporting); formal analysis (supporting); funding acquisition (supporting); investigation (supporting); methodology (supporting); project administration (supporting); resources (supporting); software (supporting); supervision (supporting); validation (supporting); visualization (supporting); writing – original draft (supporting); writing – review and editing (supporting).

## CONFLICT OF INTEREST

The authors have stated explicitly that there are no conflicts of interest in connection with this article.

## ETHICS STATEMENT

This study has been approved by the appropriate institutional review boards and has abided by ethical standards regarding studies involving human participants. All persons gave their informed consent for the use of patient data prior to inclusion in this study.

## DISCLAIMER

The content is solely the responsibility of the authors and does not necessarily represent the official views of the National Institutes of Health.

## Data Availability

The data that support the findings of this study are available from the corresponding author upon reasonable request.
